# Using Markov chain model to evaluate medical students’ trajectory on progress tests and predict USMLE step 1 scores---a retrospective cohort study in one medical school

**DOI:** 10.1186/s12909-021-02633-8

**Published:** 2021-04-09

**Authors:** Ling Wang, Heather S. Laird-Fick, Carol J. Parker, David Solomon

**Affiliations:** 1 Department of Medicine, Michigan State University, 909 Wilson Rd, 120 West Fee Hall, East Lansing, MI 48824 USA; 2 Office of Medical Education Research and Development (OMERAD), Michigan State University, East Lansing, MI USA

**Keywords:** Longitudinal study, Markov chain model, Progress tests, USMLE step 1 performance

## Abstract

**Background:**

Medical students must meet curricular expectations and pass national licensing examinations to become physicians. However, no previous studies explicitly modeled stages of medical students acquiring basic science knowledge. In this study, we employed an innovative statistical model to characterize students’ growth using progress testing results over time and predict licensing examination performance.

**Methods:**

All students matriculated from 2016 to 2017 in our medical school with USMLE Step 1 test scores were included in this retrospective cohort study (*N* = 358). Markov chain method was employed to: 1) identify latent states of acquiring scientific knowledge based on progress tests and 2) estimate students’ transition probabilities between states. The primary outcome of this study, United States Medical Licensing Examination (USMLE) Step 1 performance, were predicted based on students’ estimated probabilities in each latent state identified by Markov chain model.

**Results:**

Four latent states were identified based on students’ progress test results: Novice, Advanced Beginner I, Advanced Beginner II and Competent States. At the end of the first year, students predicted to remain in the Novice state had lower mean Step 1 scores compared to those in the Competent state (209, SD = 14.8 versus 255, SD = 10.8 respectively) and had more first attempt failures (11.5% versus 0%). On regression analysis, it is found that at the end of the first year, if there was 10% higher chance staying in Novice State, Step 1 scores will be predicted 2.0 points lower (95% CI: 0.85–2.81 with *P* < .01); while 10% higher chance in Competent State, Step 1scores will be predicted 4.3 points higher (95% CI: 2.92–5.19 with *P* < .01). Similar findings were also found at the end of second year medical school.

**Conclusions:**

Using the Markov chain model to analyze longitudinal progress test performance offers a flexible and effective estimation method to identify students’ transitions across latent stages for acquiring scientific knowledge. The results can help identify students who are at-risk for licensing examination failure and may benefit from targeted academic support.

**Supplementary Information:**

The online version contains supplementary material available at 10.1186/s12909-021-02633-8.

## Background

Medical education has evolved from a focus on the process of education to a focus on outcomes and demonstration of competence. This shift is, in part, founded on the work of Stuart E. Dreyfus and Hubert L. Dreyfus. They developed a model of skill acquisition through formal instruction and practice [1]. The Dreyfus model proposes that a student passes through five distinct stages: novice, competence, proficiency, expertise, and mastery. Modeling growth as changes in developmental stages has proven to be useful in many fields. Examples include Piaget’s stages of cognitive development [2], Kohlberg’s stages of moral development [3], stage-sequential models for reading development [4] and paired associate learning [5]. Progress testing assesses learner growth over time through the administration of examinations of similar content and difficulty across the curriculum. In 2016 our medical school adopted an innovative use of the National Board of Medical Examiners (NBME) Comprehensive Basic Science Examination (CBSE) and Customized Assessment Services (CAS) tests for progress testing twice per semester for the five semesters of the pre-clerkship curriculum. Minimum expectations for examination performance are established for each semester. The examinations contribute to students’ grades and inform decisions about progression within the curriculum. However, methodological issues may limit the generalizability of progress tests to larger scale contexts, and their ability to predict future performance in USMLE step examinations.

Previous studies correlated scores on each iteration of a progress test with USMLE Step1 results independently [6, 7] and found that later progress tests’ scores were highly correlated with Step 1 performance. In these studies, the growth paths of performance on the progress tests were ignored. Another branch of studies modelled the growth of medical knowledge using progress tests [8], but the growth of medical knowledge was not used to predict USMLE Step 1 results. Thus, it was unclear to medical educators how to best use the tests to confirm the effectiveness of the curriculum and predict student performance on the USMLE Step 1.

In this study we employed Markov chain methodology [9, 10] to evaluate medical students’ dynamic trajectories on NBME CBSE and CAS examinations given as progress tests to predict their USMLE Step 1 performance. in contrast to traditional ANOVA models, the Markov chain model considers the correlation of the previous state to the next one, naturally generating each student’s growth pattern based on estimated steady-states. This is in contrast to Growth Mixture Modeling (GMM) [11], another approach to modeling growth over time, which estimates subgroup, not individual, growth patterns. These individual growth patterns, in turn, can be used to predict Step 1 performance parametrically. The Markov chain approach to assessing growth in medical knowledge can be described as moving through several different states of knowledge as proposed by Dreyfus model. In the beginning, students have limited knowledge of medicine despite completing prerequisite science courses, and hence their performance is expected to be well below expectations for passing USLME Step 1. This can be modeled by means of a **Novice state**, in which the probability of providing a correct answer is low. At the end of a course of study, students have attained a depth of medical knowledge, and hence having a very high probability of passing USMLE Step 1, which is called **Competent state**. Depending on their learning strategies, students may pass through several intermediate states, dubbed **Advanced Beginner states**, in which they have a growing but incomplete medical knowledge base. The number of latent states and the thresholds of each latent state can be estimated by the Latent Markov model [12]. We hypothesized that students with higher transition probabilities to the Competent state would have better performance on USMLE Step 1, which is the primary outcome of the study. This study has three aims: (1) to identify the latent stages medical students go through in the first 2 years of medical school using progressive tests results, (2) to identify students’ transition probabilities among different stages, and (3) to predict USMLE Step 1 results based on their transition probabilities.

## Methods

### Student sample and measurement instruments

The sample was comprised of all medical students who matriculated to Michigan State University College of Human Medicine in Fall 2016 or Fall 2017 and finished Step 1 at the end of the second year of their program (*N* = 358). NBME progress tests were administered twice per semester for five semesters. Thus, up to ten NBME test scores per student were collected for this cohort. Most students (86.2%) completed all ten NBME tests. Missing NBME test scores were imputed using multiple imputation method [13]. We also controlled for students’ Medical College Admissions Test (MCAT) scores in the analysis, as prior studies have shown an association between MCAT and Step 1 scores [14, 15]. Linked, deidentified data were obtained using our college’s honest broker (https://omerad.msu.edu/research/honest-broker-for-educational-scholarship), a methodology previously determined to be exempt by the Michigan State University Human Subjects Review Program.

### NBME progress tests and USMLE step 1 results

The CBSE is a 200-item multiple-choice question (MCQ) exam made available by the NBME as a representative test blueprinted against the Step 1 content outline. Students receive scaled scores for the total test. Because there are a limited number of forms of the CBSE available at any given time, we also utilized the NBME’s Customized Assessment Services (CAS) to create examinations. These examinations were blueprinted to provide subscores for categories corresponding to disciplines integrated within our curriculum. NBME generated both total test percent correct scores and locally scaled scores for the total test and individual content areas we defined during the test construction phase. Percent correct scores from CAS tests were used for our analysis. Among the ten NBME tests, there were six CAS and four CBSE tests for the 2016 cohort and five CAS and five CBSE test for the 2017 cohort.

**Fig. 1:**
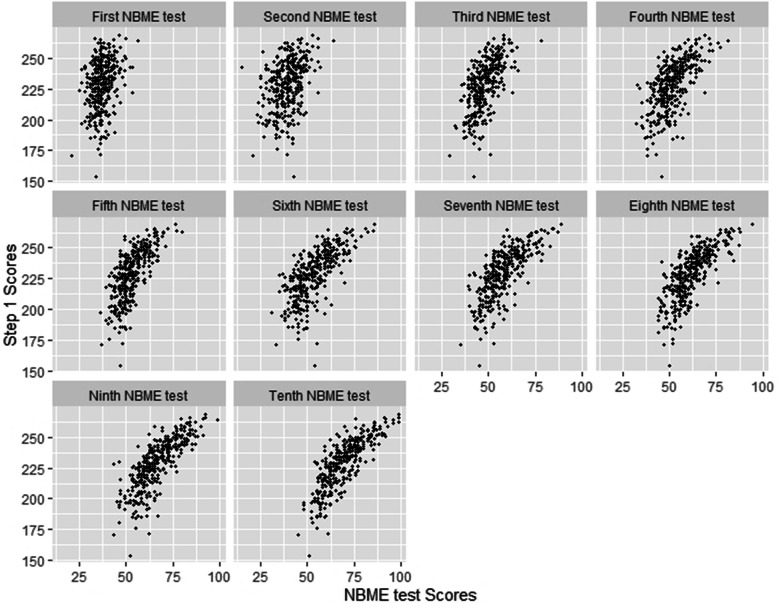
Scatter plots for NBME test versus Step 1 results

The USMLE Step 1 is a large multiple choice question (MCQ) exam given nationally to medical students to test their basic science and, to some extent, clinical knowledge. Total score is reported, with a maximum of 300 and a minimum passing score of 194 established as of January 1, 2018. Step 1 scores on first attempts in the cohort ranged from 154 to 269, with mean score 230.5. A total of seventeen students failed (score < 194). Figure 1 shows the scatterplots between each iteration of NBME test versus Step 1 results.

### Statistical Analysis

Markov chain is a stochastic model describing a sequence of possible events in which the probability of each event depends on the state attained in the previous event. To describe a Markov chain, we first define a set of states, *S =* {*s*_*1*_*,s*_*2*_*,..., s*_*r*_} where *r* indicates the number states at one time point. The process starts with one of the states and moves successively to the same state or another one at the next time point. The probabilities of moving from state *s*_*i*_ to *s*_*j*_ are called transition probabilities.

We first identified the number of latent states and the thresholds for each state using the Latent Markov Model [2]. Grid search method was employed to find the best model fit using three to six latent states. The model likelihood for each latent state was obtained based on maximum likelihood estimators. The two most commonly used criteria [Akaike information criterion (AIC) and Bayesian information criterion (BIC)] [16] were used to find the optimal number of latent states. The cutoffs of NBME test scores in each latent state were obtained based on estimated mean and standard deviation of scores in each state. A detailed setup and estimation algorithm of this Latent Markov Model is shown in the Appendix.

Once the number of latent states and score ranges for each latent stage were identified, the transition probabilities to each state were calculated for each student. Students’ steady-states were estimated based on their transition probabilities. The steady state of the Markov chain is the probability distribution of each of the equilibrium states in the long run. In our study, a student’s steady state is the likelihood (i.e. predicted probability) of the student attaining each latent state in the long run. If a student has a high likelihood of remaining in the Novice state, that suggests that the student has not been able to acquire, consolidate or apply knowledge, presumably because of inadequate learning strategies. Lastly students’ probability distributions in steady-states were regressed against their Step 1 results to see if performance on progress tests predicted Step 1 scores.

Two scenarios were considered in this study: 1) steady-states obtained from the first six NBME tests were examined to determine whether it is possible to identify academically at-risk students at the end of the first year, and 2) steady-states obtained from all ten NBME tests were examined to determine if it is possible to identify students at risk of not passing Step 1. All statistical analyses were performed using R version 3.5.3.11 [17].

## Results

### Latent states

Table 1 shows the results of the model fit using different numbers of latent states. Based on the BIC criterion, four latent states gave the lowest BIC values and provided the optimal model fit: *s*_*1*_: Novice state; *s*_*2*_: Advanced Beginner I state, *s*_*3*_: Advanced Beginner II state, and *s*_*4*_: Competent state. The range of NBME test scores in each state were *s*_*1*_: [0, 44], *s*_*2*_: (44, 56], *s*_*3*_: (56, 69] and *s*_*4*_: (69, 100].

**Table 1:** Selection of number of latent states using Latent Markov Model

Number of Latent States	Test score ranges in each latent state	AIC^a^	BIC^b^
**3**	[0,45], (45,59), (59,100]	25,851.63	26,014.38
**4**	[0, 44], (44, 56], (56, 69], (69, 100]	25,875.68	25,991.92
**5**	[0, 44], (44, 55], (56, 62], (62, 71], (71, 100]	26,197.04	26,243.54
**6**	[0, 43], (43, 50], (50, 56], (56, 64], (64, 72], (72,100]	25,928.72	26,006.22

^a^AIC equals − 2* loglikehood + 2* number of parameters

^b^BIC equals − 2* loglikehood + log (number of observations)* number of parameters

### Prediction of USMLE step 1 based on first six progress tests

**Fig. 2:**
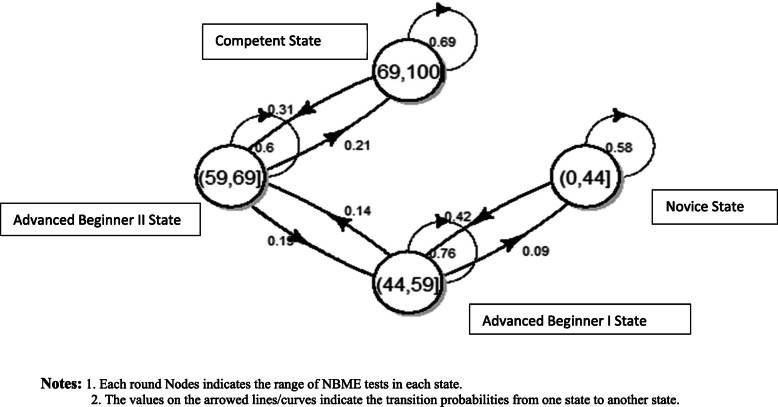
Transition probablities for all students based on first ten NBME tests. Notes: 1. Each round Nodes indicates the range of NBME tests in each state. 2. The values on the arrowed lines/curves indicate the transition probabilities from one state to another state

Students’ transition probability based on the first six NBME tests are displayed in Fig. 2. Students with NBME scores in the category of Novice state (*s*_*1*_) have a 58% of chance staying in the same state and 42% of chance improving to Advanced Beginner I state. Students in Advanced Beginner I state have a 76% chance staying in the same state, 14% chance improving to Advanced Beginner II state, and 9% chance of degrading to the Novice state. Students in Advanced Beginner II State have a 60% chance staying in the same category, 21% chance of improving, and 19% chance of degrading. The students in the Competent state have a 69% chance of staying in the same category and 31% chance of degrading at the end of the first year of medical school.

**Fig. 3:**
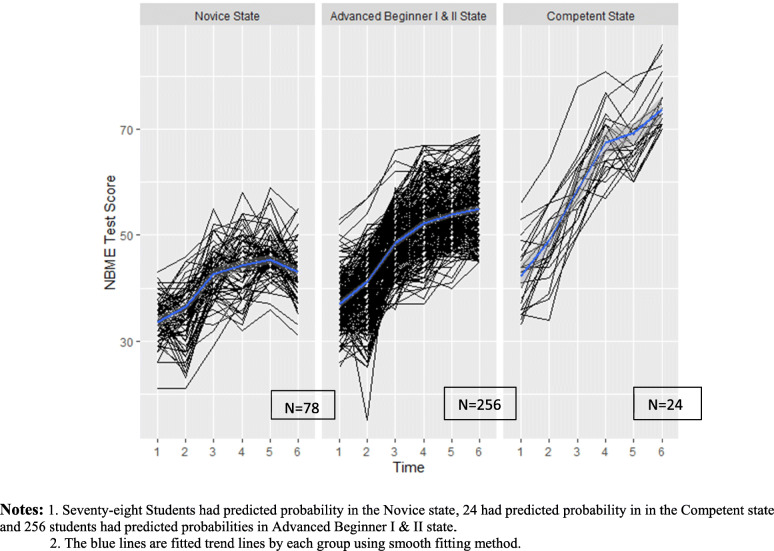
Spaghetti plot of six NBME test Scores grouped by students’ predicted probabilities in the latent states. Notes: 1. Seventy-eight Students had predicted probability in the Novice state, 24 had predicted probability in in the Competent state and 256 students had predicted probabilities in Advanced Beginner I & II state. 2. The blue lines are fitted trend lines by each group using smooth fitting method

Based on the transition probabilities, the predicted probability in each state at the end of first year in medical school for all 358 students were: 8.5% Novice, 39.9% Advanced Beginner I, 30.4% Advanced Beginner II, and 21.2% Competent state. Each student’s predicted probability in each state was estimated based on their own transition probability. Figure 3 shows the spaghetti plot of six NBME tests results grouped by the students’ predicted probability in each state, with Advanced Beginner I and II states combined. We combined Advanced Beginner I and II states because the spaghetti plots of these two states didn’t have strong differences. Among the 78 students who had predicted probability in the Novice state at the end of first year, the mean Step 1 score was 209 (SD = 14.8), and 7 failed Step 1 on the first attempt. On the other hand, 24 students had predicted probability to be in the Competent state at the end of first year, their mean Step 1 score was 255.1 (SD = 10.8).

Next the predicted probability distribution in each state were regressed on students’ Step 1 results, controlling for MCAT scores. The regression results show that if the probability of steady-states in the Novice state increased by 10%, the predicted Step 1scores would be 2.0 points lower (95% CI: 0.85–2.81 with *P* < .01); 10% higher probability of steady-states in Advanced Beginner I state led to 0.3 points lower on Step 1 (95% CI: − 0.67-1.02 with *P* = .53); 10% higher probability of steady-states in Advanced Beginner II state led to 2.7 points higher on Step 1 (95% CI: 1.03–3.21 with *P* < .01); and 10% higher probability in the Competent state lead to 4.3 points higher on Step 1 (95% CI: 2.92–5.19 with *P <* .01). No one in this final group failed Step 1.

### Prediction of USMLE step 1 based on ten progress tests

**Fig. 4:**
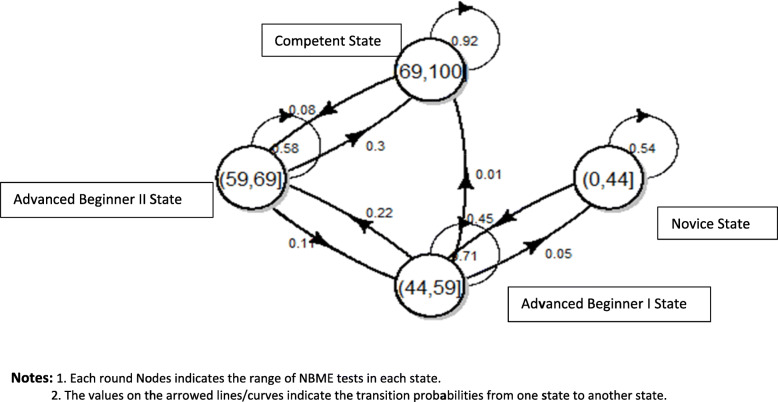
Transition probablities for all students based on all 10 NBME tests. Notes: 1. Each round Nodes indicates the range of NBME tests in each state. 2. The values on the arrowed lines/curves indicate the transition probabilities from one state to another state

The same analysis was replicated using all ten iterations of NBME tests. The transition probabilities of all students are displayed in Fig. 4. Compared with the transition probability based on the first six NBME tests, students had a lower probability of degrading from Competent state to Advanced Beginner II (8%) and a higher probability of staying in the Competent Learned State (92%). The predicted probability for each state at the end of the second year for all students was: 1.1% Novice, 8.9% Advanced Beginner I, 18.3% Advanced Beginner II, and 71.8% for Competent state.

**Fig. 5:**
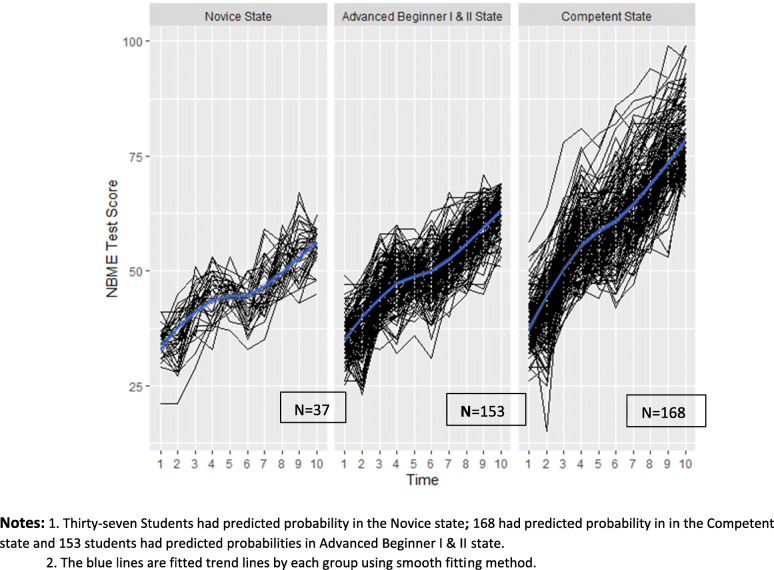
Spaghetti plot of ten NBME test scores grouped by students’ predicted probabilities in the latent states. Notes: 1. Thirty-seven Students had predicted probability in the Novice state; 168 had predicted probability in in the Competent state and 153 students had predicted probabilities in Advanced Beginner I & II state. 2. The blue lines are fitted trend lines by each group using smooth fitting method

Figure 5 shows the spaghetti plot of students’ ten NBME tests by their predicted probability in the latent states. There were four students who had a greater than 50% chance of staying in the Novice state. Among these four students, two (50%) failed Step 1 and one (25%) barely passed (score = 195). Eighty-four students still had a high probability of being in the Novice or Advanced Beginner I state. Their mean Step 1 score was 209 (SD = 18.6), and 15 (18%) of them failed Step 1. One hundred and sixty-eight students had predicted probability to be in the Competent state. Their mean Step 1 score was 243.3 (SD = 11.6) and none of them failed Step 1.

These regression results are similar to those from our six test model: a 10% higher chance in the Novice state led to scoring 3.0 points lower on Step 1 (95% CI: 2.23–5.45 with *P* < .01); 10% higher chance in Advanced Beginner I state led to scoring 0.6 points lower (95% CI: − 0.78-1.04 with *P* = 0.11); 10% higher chance in Advanced Beginner II state led to scoring 1.3 points higher (95% CI: 0.33–2.06 with *P <* .01); and 10% higher chance in the Competent state led to scoring 3.4 points higher (95% CI: 2.18–4.28 with *P <* .01).

## Discussion

Though progress tests have been used for decades in medical education, most programs suffer from methodological limitations which limit generalizability to other contexts. In this study, we introduce an innovative assessment method to model students’ progression in acquiring basic medical knowledge using NBME examinations administered as progress tests. The standard methodological framework for the study of intra-individual differences’ change over time in continuously measured variables is growth curve modeling [18–22]. Growth curve modeling takes as its data source individual empirical growth trajectories. Growth curve modeling can provide an estimate of the average initial level and average rate of growth taken to be estimates of the growth parameters in a defined population. Compared to growth curve methods, Markov chain method not only considers the correlation between the previous and next test performance but also provides an estimated probability distribution of an individual’s growth pattern which can be used to predict high-stake test performance, such as on Step 1.

Employing the Markov chain method, we can find each student’s transition probability among four stages and their predicted probability in each stage. The regression results show that the students with higher probabilities of staying in the lowest state (Novice) will have significantly lower Step 1 performance, while those with higher probabilities for attaining the Competent state will have significant higher Step 1 results. Based on the transition probability, we can identify students with little growth during the first year of medical school (i.e. the first six examinations) and provide additional academic support to them to enhance their trajectory in the second year.

Our study has two important limitations. First, this study focused only on one medical school and two student cohorts within this medical school. Our school implemented a new competency-based, integrated curriculum in 2016 which may impact our students’ trajectories for learning. Our entering students’ characteristics, however, were similar to that for other US medical schools (Mean MCAT is 506.2 for these two cohort students). Our model of progress testing twice per semester is relatively unique in the U.S., but could be readily implemented in other schools. Progress testing had a long history in countries such as Netherlands where medical schools collaborate and share a larger pool of items resulting a cost reduction and shared benchmarking [23]. Future studies are necessary to assess how well our findings might generalize to other student cohorts and medical schools. Second, we used two different NBME exam types for our progress testing. Blueprinting for content and overall item difficulty was similar, but not identical, between the two types. The NBME reports scaled scores for the CBSE, normed to a mean of 70 with a standard deviation of 8 for first time Step 1 takers. In contrast, CAS scores are reported as percentage correct. The two types of scores tracked quite well with one another, but they cannot be equated.

With these caveats in mind, this study significantly contributes to our understanding of progress testing in at least two areas. First, it has provided strong validity evidence for our internal assessment program and provides an estimation method for faculty members to gauge the progress of students and intervene with additional academic support as needed. Second, the analytical approach proposed in this study provides a flexible method by which medical knowledge growth can be categorized into latent states. It can provide useful information for medical educators interested in pursuing progress examinations, even after the USMLE Step 1 changes to Pass/Fail.

## Conclusion

In this study, four latent growth patterns of medical students acquiring basic medical knowledge were identified based on NBME examinations administered progress tests in one medical school. In the future study, we will incorporate more cohort students from our school or other medical schools to examine whether similar growth patterns can be identified. Also, characteristics of students in each growth pattern will be examined to identify factors leading to different trajectories.

## Supplementary Information

**Additional file 1.**

## Data Availability

De-identified data set can be available upon requests.
