# Supplementary material for: Using Markov chain model to evaluate medical students’ trajectory on progress tests and predict USMLE step 1 scores---a retrospective cohort study in one medical school
Source: BMC Med Educ. 2021 Apr 9;21:200. doi: 10.1186/s12909-021-02633-8 (PMC8033658; doi:10.1186/s12909-021-02633-8)
Supplement: Supplementary file 1 — Additional file 1. [file 12909_2021_2633_MOESM1_ESM.docx]

**Appendix: Setup and Estimation of Latent Markov Model in the Study**

The setup of Latent Markov model can be described as

$$f^{\left( t \right)}\left( y | u,x \right)=f_{Y^{(t)}|U^{\left( t \right)},X^{\left( t \right)}}\left( y | u,x \right)$$

$$t=1,\ldots,T; u=1,\ldots,k$$

$Y^{(t)}$ are the NBME test results at time $t$ , $u$ is the latent states and $X^{(t)}$ are the covariates at time $t$.

The initial probabilities

$$\pi_{u}=f_{U^{\left( 1 \right)}}\left( u \right), u=1,\ldots,k$$

And the transition probabilities are

$$\pi_{u|\bar{u}}^{(t)}=f_{U^{\left( t \right)}|U^{\left( t-1 \right)}}\left( u|\bar{u} \right)$$

Where $\bar{u}$ is a realization of $U^{\left( t-1 \right)}$.

The log-likelihood of Latent Markov model can be expressed as

$$l\left( \theta\right)=\sum_{i=1}^{n} logf_{Y|X}\left( y | x \right)=\sum_{t=1}^{T} \sum_{u=1}^{K} \sum_{x} \sum_{y} a_{uxy}^{(t)}\log\varphi^{\left( t \right)}\left( y | u,x \right)$$

$$+\sum_{u=1}^{K} \sum_{x} b_{ux}^{(1)}\log\pi_{u|x}+\sum_{t=2}^{T} \sum_{\bar{u}=1}^{K} \sum_{u=1}^{K} \sum_{x} b_{\bar{u}ux}^{(t)}\log\pi_{u|\bar{u},x}^{(t)}$$

Where $b_{ux}^{(1)}$ is the frequency of the latent state $u$ and covariate $x$ at time $t$; $b_{\bar{u}ux}^{(t)}$ is the number of transitions from state $\bar{u}$ to state $u$, whereas $a_{uxy}^{(t)}$ is the frequency of subjects that in latent state $u$ and provide response $y$. We assume that $\varphi^{\left( t \right)}\left( y | u,x \right)=\frac{1}{\sqrt{2\pi\sigma^{2}}}exp\left[ -1/2\frac{{(y-\tau_{ux}^{\left( t \right)})}^{2}}{\sigma^{2}} \right]$

To obtain the Maximum Likelihood Estimator (MLE) $\hat{\theta}$ (including $\pi_{u}, \pi_{u|\bar{u},x}^{(t)}$and $\varphi\left( y | u,x \right)$), Expectation-Maximum (EM) algorithm is employed: E-step computed expected value of the $a_{uxy}^{(t)}$, $b_{\bar{u}ux}^{(t)}$ and $b_{ux}^{(t)}$ and M-step update $\hat{\theta}$ by maximizing the expected value of $l\left( \theta\right)$ based on expected parameters obtained from E-step. Two steps are alternated till convergence to find $\hat{\theta}$.
